# Intact survival from severe cardiogenic shock caused by the first attack of atrial tachycardia treated with extracorporeal membrane oxygenation and surgical left atrium appendage resection: a case report

**DOI:** 10.1186/s40981-021-00481-5

**Published:** 2021-11-03

**Authors:** Tatsuhiko Shimizu, Tomoyuki Kanazawa, Takanobu Sakura, Kazuyoshi Shimizu, Tatsuo Iwasaki

**Affiliations:** Department of Anesthesiology and Resuscitology, Okayama University Graduate School of Medicine, Dentistry and Pharmaceutical Sciences, 2-5-1 Shikata-cho, Kita-ku, Okayama, 700-8558 Japan

**Keywords:** Focal atrial tachycardia, Central extracorporeal membrane oxygenation, Surgical ablation

## Abstract

**Background:**

Atrial tachycardia (AT) is rare in children and can usually be reversed to sinus rhythm with pharmacotherapy and cardioversion. We report a rare case of severe left-sided heart failure due to refractory AT.

**Case presentation:**

A 12-year-old boy had AT with a heart rate of 180 beats/minute, which was refractory to any medication and defibrillation despite the first attack. Due to rapid cardiorespiratory collapse shortly after arriving at our hospital, central extracorporeal membrane oxygenation (ECMO) with left arterial venting was started immediately. Although AT persisted after that, it stopped on the 3rd day after admission following surgical resection of the left atrial appendage thought to be the source of AT. He was weaned off ECMO on the 7th day and ventilator on the 14th day.

**Conclusions:**

The appropriate timing of central ECMO and surgical ablation were effective in saving this child from a life-threatening situation caused by refractory AT.

## Background

Atrial tachycardia (AT) is one of the rare diseases in pediatric patients [1]. AT attack is treated by antiarrhythmic medication such as adenosine triphosphate (ATP), amiodarone, and β-blocker. Some patients have refractory AT that does not respond well to usual medical treatment. Long-lasting AT may cause cardiomyopathy when medical treatment would not effect for a long time. Extracorporeal membrane oxygenation (ECMO) support is occasionally necessary for hemodynamic support in severe cardiogenic shock with refractory AT [2]. Catheter ablation is one of the treatments for AT [3], especially when medication would not be effective. Furthermore, surgical resection of AT focus may be considered after failed catheter ablation [4, 5]. We experienced a child who had a severe cardiogenic shock with the first episode of AT attack. We were able to rescue him with ECMO support and surgical ablation.

## Case presentation

**Fig. 1:**
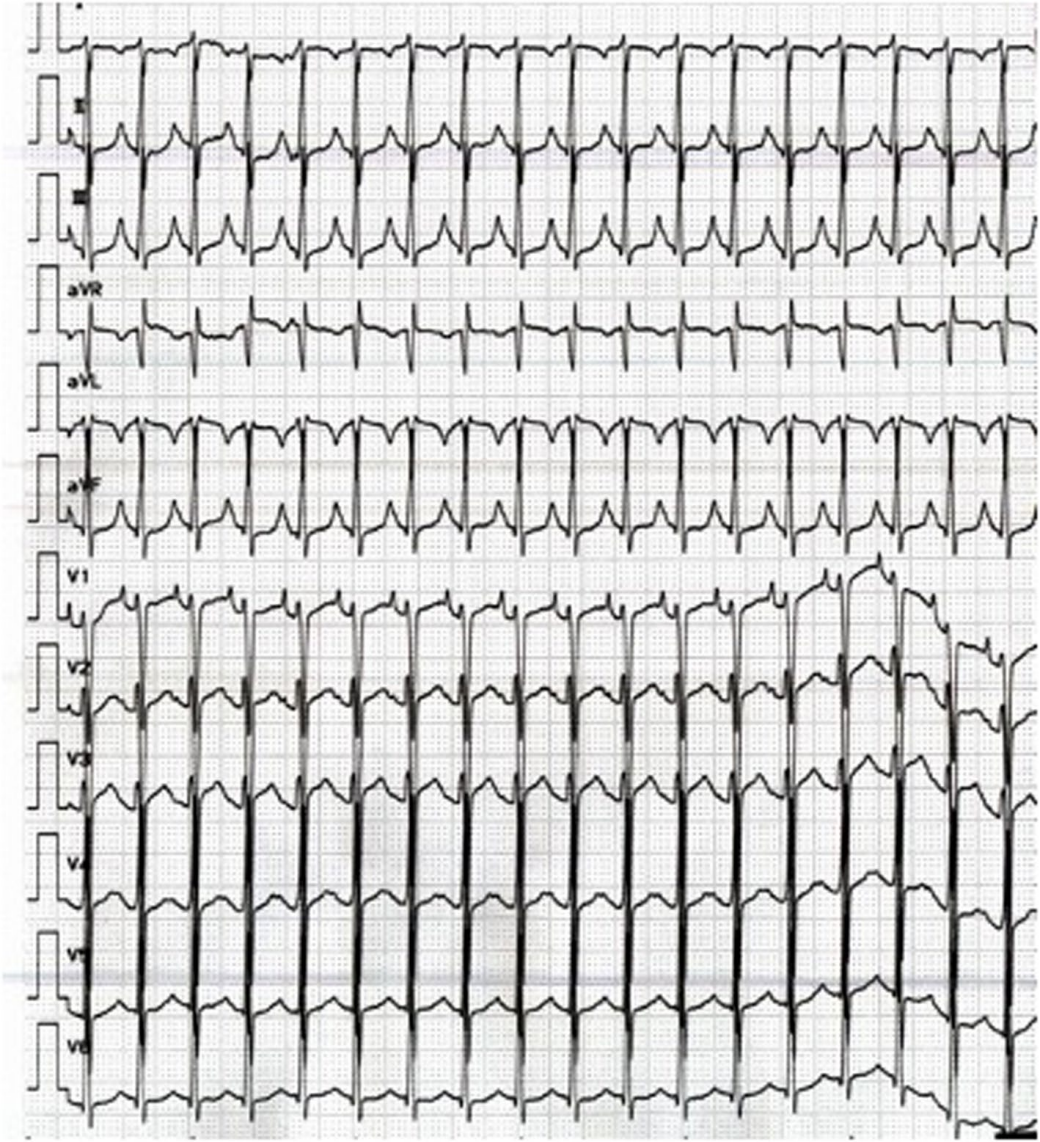
Electrocardiogram on admission to ICU. Regular positive *P* waves of 180–190 bpm and corresponding narrow QRS waves are seen in V1 and V2, although the *P* wave is masked by the *T* wave in most leads.

**Fig. 2:**
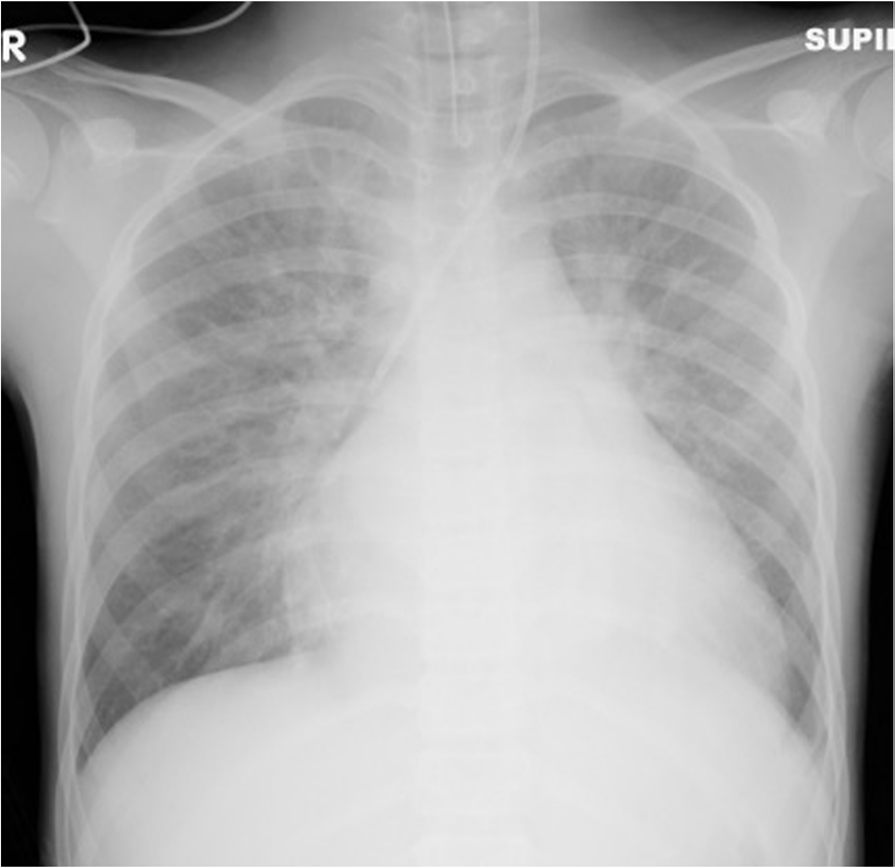
Chest radiograph on admission to ICU

**Fig. 3:**
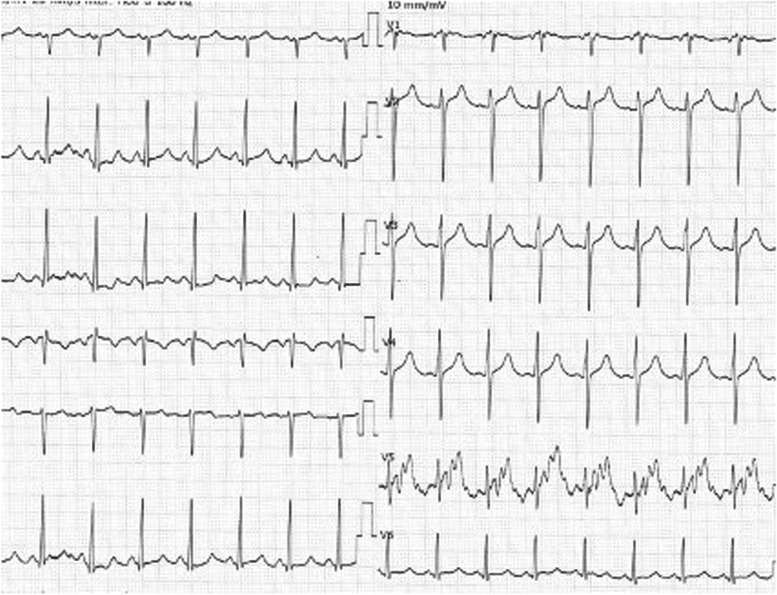
Electrocardiogram after surgical ablation

A 12-year-old boy (height 145 cm, weight 33 kg) was diagnosed with AT of more than 170 beats per minute (bpm) at the first visit to a clinic. He had felt bad feelings for several days without any common cold symptoms. He was transported to our hospital by car with his family. At the emergency department (ED), he was alert, but his heart rate (HR) was 180bpm and his systolic blood pressure (sBP) was 80 mmHg. The initial ECG showed a narrow complex tachycardia (Fig. 1), and transthoracic echocardiography showed only 20% of left ventricular ejection fraction, suggesting cardiomyopathy [1]. We suspected tachycardia-induced cardiomyopathy rather than acute myocarditis based on the blood tests of CRP 0.08 mg/dL, WBC 6240 /μL, CK 363 U/L, and CK-MB 14 U/L, no ST changes on ECG, and no pericardial effusion on echocardiography. His respiratory status was stable and SpO_2_ was maintained 100% in room air. We started amiodarone-loading 5mg/kg intravenously, and kept continuous infusion at 10 μg/kg/min, following three times ATP administration (10mg, 15mg, 20mg) and twice of electric cardioversion (20J, 30J), which did not have any improvement. During the medical treatment at ED, sBP was going down around 60 mmHg, and SpO_2_ was 90–96% on oxygen mask (100% O_2_, 4 L/min). His hemodynamic and respiratory status had been worsening at ED, we decided to transport him to the intensive care unit (ICU) after endotracheal intubation. After ICU admission, we inserted an arterial line via the right brachial artery and a central venous line via the left jugular vein. We started milrinone infusion at 0.2 μg/kg/min and noradrenaline at 0.02 μg/kg/min. Subsequently, his sBP improved from 50 to 80 mmHg; however, AT with HR of 180–200 bpm was not improved. Therefore, we decided to establish veno-arterial (VA) ECMO support for hemodynamic and respiratory instability due to left ventricular failure leading to pulmonary edema (Fig. 2). We chose central ECMO rather than peripheral ECMO to ensure complete unloading left ventricle by inserting the left atrial venting cannula. Central VA ECMO via median sternotomy was uneventfully performed using 24-Fr right atrial, 20-Fr left atrial, and 16-Fr aortic cannula under general anesthesia with sevoflurane 1–1.5%, fentanyl 500 μg, and rocuronium in the operating room (OR). Under 150 ml/kg/min of ECMO flow support, we continued to provide amiodarone infusion up to 20 μg/kg/min and started continuous landiolol infusion up to 10 μg/kg/min. His HR was decreased to 140–160 bpm several hours after ECMO induction and was settled down to 100–140 bpm on the 2nd clinical day; however, his cardiac rhythm was still AT. Although there was bleeding of 100–150 ml per hour under the control of ACT for around 150 s on the 2nd clinical day, the bleeding gradually settled down with blood transfusion as needed, and he underwent removal of intrathoracic hematoma in OR on the 4th clinical day. A pediatric cardiologist had diagnosed that the focus of his AT could be the left atrial appendage (LAA), based on ECG findings that the polarity of the *P* wave was positive on V1, negative on I, positive on II, III, and aVF. Immediately after the cardiac surgeon clamped the base of the LAA according to the cardiologist’s opinion, the patient’s cardiac rhythm was reverted to sinus rhythm around 50–60 bpm. The surgeon resected LAA as a surgical treatment of AT. After the surgical treatment, this child did not have an episode of AT during the ICU stay (Fig. 3) and we could completely discontinue amiodarone and landiolol infusion until the 5th clinical day. His cardiac function was dramatically improved, and we finally discontinued ECMO support on the 7th clinical day and milrinone on the 20th clinical day. He was extubated on the 14th clinical day. He was transported to the general ward without any antiarrhythmic medications on the 30th clinical day.

## Discussion

We found two unique clinical viewpoints in this case. The first is the boy had been on central ECMO support for a sudden worsening of hemodynamic and respiratory status due to cardiomyopathy caused by refractory AT despite the first attack, and the second is he underwent surgical resection of LAA to treat AT on ECMO support.

Atrial tachycardia is rare in children. The median age at diagnosis is 7.2 years with the most commonly occurring from birth to 1 year, and most patients are treated with antiarrhythmic medications (ATP, amiodarone, β-blocker), cardioversion or catheter ablation [1]. Antiarrhythmic agents are well effective to recover normal sinus rhythm in most cases [1]; however, AT may cause cardiomyopathy when arrhythmic episode repeats frequently. When medical treatments as above would not be effective, mechanical hemodynamic support by ECMO should be considered for a rescue therapy because AT may cause cardiomyopathy when AT would not be recovered to sinus rhythm for a certain period of time [2, 6]. Remarkably decreased left ventricular ejection fraction suggested cardiomyopathy, which required prompt treatment in this child. Several pediatric cases showed that ECMO support was performed for cardiomyopathy associated with refractory AT [1, 2] and refractory arrhythmias [2, 6–8]. There have been no reports of patients requiring ECMO within a few days after the first attack. In this case, he had already had a severe cardiac failure on arrival at ED, which dramatically progressed leading to pulmonary edema despite treatment such as amiodarone infusion, bolus of ATP, and electric cardioversion. Our choice to induce ECMO as a rescue therapy for refractory AT was considered to be reasonable in this case. If we had hesitated to introduce ECMO, cardiac arrest and neurological sequelae would have been more likely to occur. In fact, Kang et al. reported a case of a 13-year-old girl who died of serious neurological complications after requiring ECMO due to circulatory collapse during the administration of amiodarone for treating refractory AT [1]. We also selected central ECMO with thoracotomy instead of peripheral ECMO. Although peripheral ECMO via inguinal approach does not require a thoracotomy, it not only increases left ventricular afterload, but also exacerbates pulmonary edema and delays recovery of left ventricular function due to inadequate left atrium (LA) decompression [9]. In contrast, central ECMO solves these problems, and Kotani et al. reported that ECMO with early LA decompression increases the rate of recovery of lung and left ventricular function [9]. After all, the establishment of ECMO before the collapse contributed to the neurological prognosis, and the choice of central ECMO, which is possible to drainage blood from both atriums, instead of peripheral ECMO may have resulted in a faster weaning from ECMO and ventilator because of avoiding the damage to the heart and lungs [9]. Although bleeding complications are a greater problem with central ECMO than with peripheral ECMO, we think that the advantages of central ECMO outweighed the disadvantages of hemorrhage since the left ventricular function was significantly impaired in this case and peripheral ECMO could have worsened the condition of the heart and lungs due to increased left atrial and ventricular pressure with increased afterload.

In the second unique situation, surgical ablation was performed under ECMO support in this case. As a therapeutic option, catheter ablation is considered in the case of refractory AT, but it was difficult to perform catheter ablation in this case because of the rapid deterioration of his condition. Previous reports have shown that the atrial appendage is the source of focal AT in 11% of cases [1]. For refractory AT of LAA origin, Khan et al. reported a successful case of radiofrequency ablation on ECMO support [2], but Phillips et al. described that catheter ablation of focal AT resulting from LAA, especially distal point, was difficult due to the complex anatomy of LAA, and surgical appendectomy or thoracoscopic surgery may be required [10]. Pokushalov et al. reported a case of hemodynamically stable that ineffective catheter ablation followed by successful surgical ablation [4]. In this case, the chest had already been opened for central ECMO, and it was reasonable to perform surgical ablation during ECMO support as a rescue treatment because it was very difficult to plan catheter ablation in this situation.

Finally, he was completely recovered from hemodynamic and respiratory failure by multidisciplinary treatment. ECMO support and surgical ablation played the main role in treating AT.

## Conclusion

We had a rare case that the boy required ECMO support for severe hemodynamic and respiratory failure caused by refractory AT on the first attack. Adequate timing of ECMO induction and surgical treatment was effective to save children from a life-threatening situation caused by refractory AT.

## Data Availability

Not applicable.

## Abbreviations

AT: Atrial tachycardia
ECMO: Extracorporeal membrane oxygenation
ATP: Adenosine triphosphate disodium hydrate
bpm: Beat per minute
ED: Emergency department
HR: Heart rate
sBP: Systolic blood pressure
ICU: Intensive care unit
VA: Veno-arterial
OR: Operating room
LAA: Left atrial appendage
LA: Left atrium
